# Testing sea urchin and green sea turtle consumption of the allelopathic macroalga *Galaxaura divaricata*

**DOI:** 10.1002/ece3.11324

**Published:** 2024-04-25

**Authors:** Carolin Nieder, Siobhan Jean Heatwole, Chen‐Pan Liao, Chen‐Lu Lee, Chaolun Allen Chen, Shao‐Lun Liu

**Affiliations:** ^1^ Institute of Marine Science University of Auckland, Leigh Marine Laboratory Leigh, Auckland New Zealand; ^2^ School of Earth, Atmospheric and Life Sciences University of Wollongong Wollongong New South Wales Australia; ^3^ Department of Biology National Museum of Natural Science Taichung Taiwan; ^4^ Institute of Marine Biology National Taiwan Ocean University Keelung Taiwan; ^5^ Biodiversity Research Center Academia Sinica Taipei Taiwan; ^6^ Department of Life Science and Center for Ecology and Environment Tunghai University Taichung Taiwan

**Keywords:** allelopathic, coral reefs, feeding preference, *Gracilaria edulis*, herbivory, macroalgae

## Abstract

*Galaxaura divaricata* is a partially calcified macroalga that hampers coral recruitment, growth, and recovery via the excretion of allelopathic secondary metabolites. Herbivorous fishes are not major consumers of *Galaxaura* spp. and there is a need to understand feeding preferences for *Galaxaura divaricata* in other macroherbivores, like sea urchins and green sea turtles that could act as potential controlling agents. Under certain environmental conditions, *G*. *divaricata* can proliferate and overgrow degraded reefs for several years, as documented for several coral patch reefs in the lagoon of Dongsha Atoll, South China Sea. This study aimed to experimentally test the feeding preferences of five species of sea urchin and two individual green sea turtles, *Chelonia mydas*, for *G*. *divaricata*. Specifically, we quantified and compared the consumption rates of the allelopathic *G*. *divaricata* with *Gracilaria edulis*, a nonallelopathic, fleshy red alga, known to be highly favored by herbivores. Results showed that the five urchin species fed on both *G*. *edulis* and *G*. *divaricata*. However, urchins consumed 2–8 times less wet weight of *G*. *divaricata* (range 0.3–3.1 g urchin^−1^ 24 h^−1^) compared to *G*. *edulis* (range 0.6–18 g urchin^−1^ 24 h^−1^), suggesting that urchin grazing may exert some control on *G*. *divaricata* abundance but is likely ineffective for a large‐scale removal of the alga. Further, both green sea turtles avoided *G*. *divaricata* and selectively fed on *G*. *edulis*. More experiments are needed to test the potential role of herbivores in controlling the overgrowth of coral competitive and allelopathic macroalgae, like *Galaxaura* on coral reefs.

## Graphical abstract

This study investigates the feeding preferences of sea urchins and green sea turtles for the coral‐damaging macroalga *Galaxaura divaricata*. Results indicate that while sea urchins consume both *G*. *divaricata* and a preferred nonallelopathic alga, their consumption rates are significantly lower for *G*. *divaricata*. Moreover, green sea turtles show a clear preference for the nonallelopathic alga, suggesting potential limitations in the efficacy of herbivores in controlling *G*. *divaricata* overgrowth on coral reefs, highlighting the need for further research in this area.

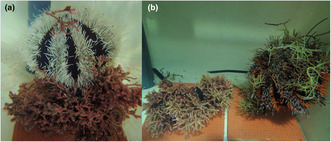

## 1 INTRODUCTION

On healthy coral reefs, algae consumption by herbivores promotes a healthy coral‐dominated state, whereby the herbivores keep the growth of algae that compete with coral in check (Edmunds & Carpenter, 2001; Hughes et al., 2007; Ishikawa et al., 2016; Steneck et al., 2017; Williams & Polunin, 2001). Coral reef fishes, sea urchins, and sea turtles are keystone macroherbivores known to be important in determining the structure and abundance of macroalgae assemblages on coral reefs (Alcoverro & Mariani, 2002; Chong‐Seng et al., 2014; Goatley et al., 2012; Steneck, 1988). For instance, sea urchin grazing was shown to be of major importance for top‐down regulation of macroalgae on Caribbean reefs, especially for those that were overfished (De Ruyter van Steveninck & Bak, 1986; Edmunds & Carpenter, 2001; Hind et al., 2019; Ishikawa et al., 2016; Liddell & Ohlhorst, 1986; Sammarco, 1982). The large‐scale die‐off of the sea urchin *Diadema antillarum* across the Caribbean in the 1980s triggered a dramatic increase in algal abundance leading to macroalgae dominance on many coral reefs (Lessios et al., 1984). The feeding of macroalgae by green sea turtles (*Chelonia mydas*) maintains algal communities in a cropped state, preventing their proliferation and expansion (Goatley et al., 2012; Wilson, 2010). A decline in herbivores and a lack of effective algae removal (top‐down control) can facilitate the proliferation of macroalgae and lead to phase shifts on coral reefs toward macroalgae dominance (Mantyka & Bellwood, 2007a, 2007b). To maintain healthy reef ecosystems and restore those in decline, a frequently suggested strategy is to increase the number of grazers to mitigate algae overgrowth on coral reefs (Steneck et al., 2017; Stimson et al., 2007; Williams & Polunin, 2001). However, for this strategy to work, it is important to understand the dietary preferences of macrograzers for dominant macroalgae.

*Galaxaura divaricata* (Linnaeus) Huisman & Townsend is a tropical, partially calcified, red macroalga, commonly found in shallow seagrass beds and on coral reefs across the Pacific Ocean (Huisman & Townsend, 1993; Kurihara et al., 2004; Wang et al., 2005). Macroalgae of the genus *Galaxaura* produce active secondary metabolites (allelochemicals) that can bleach and kill competing coral upon direct contact (Rasher et al., 2011; Rasher & Hay, 2010a). These allelochemicals also inhibit coral larval settlement (Dixson et al., 2014), hampering the recruitment, growth, and recovery of degraded coral reef ecosystems (Rasher & Hay, 2010b). Long‐term *G*. *divaricata* blooming events have recently been documented on shallow coral patch reefs in the lagoon of Dongsha Atoll (Taiwan) in the South China Sea (Nieder et al., 2019). Dongsha Atoll is the largest coral reef atoll in the northern South China Sea and serves as an important fish stock contributor and plays a role in sustaining connectivity for coral reef species in the region (Liu et al., 2021; Yu et al., 2022). However, this unique ecosystem is extremely vulnerable due to the impacts of climate change, and illegal and unregulated fishing (Liu et al., 2021). Recurring disturbances (e.g., typhoon damage, coral bleaching) combined with specific environmental conditions (e.g., shallow waters, low wave action, high nutrient load) can trigger *G*. *divaricata* to proliferate inside the Dongsha lagoon, resulting in persistent overgrowths that can last for several years (Nieder et al., 2019). The accelerating rates of biodiversity loss in Dongsha and the proliferation of allelopathic macroalgae, like *Galaxaura* on coral reefs in the lagoon (Nieder et al., 2019) have increased the need for effective management that can improve ecosystem resilience (Cheng et al., 2020; Dai, 2004; Wang et al., 2007).

*Galaxaura* is among the least preferred foods for various herbivorous fishes (Loffler et al., 2015; Mantyka & Bellwood, 2007a, 2007b). Mantyka and Bellwood (2007a) found the lowest grazing frequencies and minimal biomass removal of *G*. *filamentosa* by common herbivorous fishes compared to other macroalgae. Recent field observations further suggest that, while several species of coral reef fish feed on epiphytes and epifauna from the surface of *G*. *divaricata*, they do not readily remove *G*. *divaricata* itself (Nieder et al., 2022). Consequently, there is a need to understand whether *G*. *divaricata* is consumed by macroherbivores other than fish that could potentially control the abundance of this algae on coral reefs. However, nothing is currently known about the dietary choices of sea urchins and green sea turtles for locally abundant macroalgae, like *G*. *divaricata*.

Therefore, this study aimed to test the feeding preference of five species of sea urchins and two individual green sea turtles, *Chelonia mydas* for the allelopathic, partially calcified red alga *G*. *divaricata*. We conducted feeding assays in a captive setting to quantify and compare the consumption rates of *G*. *divaricata* and *Gracilaria edulis*, a nonallelopathic, fleshy red alga, known to be a preferred food for many marine herbivores (Garnett et al., 1985; Seymour et al., 2013).

## 2 MATERIALS AND METHODS

### 2.1 Site description

Feeding assays were carried out on sea urchins and green sea turtles at the aquarium of the Dongsha Atoll Research Station within the Dongsha Atoll Marine National Park from June to August 2016. Dongsha Atoll (also known as Pratas Island) is a large horseshoe‐shaped coral reef atoll located in the northern South China Sea (20°40′43″ N, 116°42′54″ E) that is located about 414 km directly southwest of Kaohsiung (Taiwan) and about 338 km southeast of Hong Kong. The atoll is composed of a semi‐closed lagoon (diameter about 25 km, max depth about 20 m), a small coral sand island (1.74 km^2^, known as Dongsha Island), and a wide reef flat. The lagoon has a soft sediment bottom that is interspersed with seagrass beds and numerous coral patch reefs, many of which have been severely degraded and overgrown by macroalgae (Nieder et al., 2019). Despite being a proclaimed marine protected area (MPA) of Taiwan, the Dongsha Atoll Marine National Park is subject to overexploitation and illegal, unreported, and unregulated fishing activities by fishers from countries around Taiwan, posing a serious threat to marine biodiversity (Yu et al., 2022). Dongsha Atoll provides an important nesting and feeding ground for endangered green sea turtles (*Chelonia mydas*), which have been illegally exploited in Dongsha (Figure S1). The Dongsha Marine National Park provides crucial natural habitats to protect juvenile green sea turtles that could eventually contribute to the growth and recovery of some nesting populations.

### 2.2 Study organisms

#### 2.2.1 Sea urchins

Sea urchins were collected from seagrass beds and shallow patch reef areas at 1–6 m depth. A total of 15 adult sea urchins were collected, spanning five species. Sample sizes were as follows: *Tripneustes gratilla* Linnaeus, *n* = 3, weight range: 286–342 g; *Echinothrix calamaris* Pallas, *n* = 2, weight range: 437–561 g; *Diadema savignyi* Audouin, *n* = 2, weight range: 73–120 g; *Diadema setosum* Leske, *n* = 1, weight 76 g; and *Echinometra mathaei* Blainville, *n* = 7, weight range: 16–41 g. Unfortunately, due to the shear paucity of sea urchins in the Dongsha lagoon (see sea urchin density survey data in Nieder et al., 2019), we were not able to find more individuals for the present study. The urchins were held separately in 900 L aquaria equipped with a seawater recirculation system (average water temperature: 28.8 ± 0.2°C, salinity: 35 ppm). After urchins had acclimated for 10 days, macroalgae feeding assays were carried out for 12 consecutive days. All sea urchins were released into their natural habitats after completion of the experiment.

### 2.3 Green sea turtles

Two green sea turtles, *Chelonia mydas* (Linnaeus); body length range of 70–80 cm were rescued from an illegal fishing boat by the Taiwanese Coast Guard and brought for rehabilitation to the Dongsha Atoll Marine National Park. The turtles were kept separated from each other in two circular tanks (180 and 290 cm diameter) connected to a seawater recirculation system (average water temperature 26.6°C, pH 8.66, salinity 35 ppm, dissolved oxygen 6.61 ppm). The turtles were injured and were allowed to rest in the tanks for 4 weeks prior to any experimentation to allow sufficient time for them to recover and adjust. During the acclimation period, the turtles were fed one medium‐sized squid twice a day. In addition, the turtles received one daily tray of seagrass. After the 4 weeks of acclimation, macroalgae feeding assays were carried out for 10 consecutive days. Squid meals were ceased when the algae experiment began. The turtles were released into their natural habitat after 6 weeks of rehabilitation.

### 2.4 Feeding assays

**FIGURE 1:**
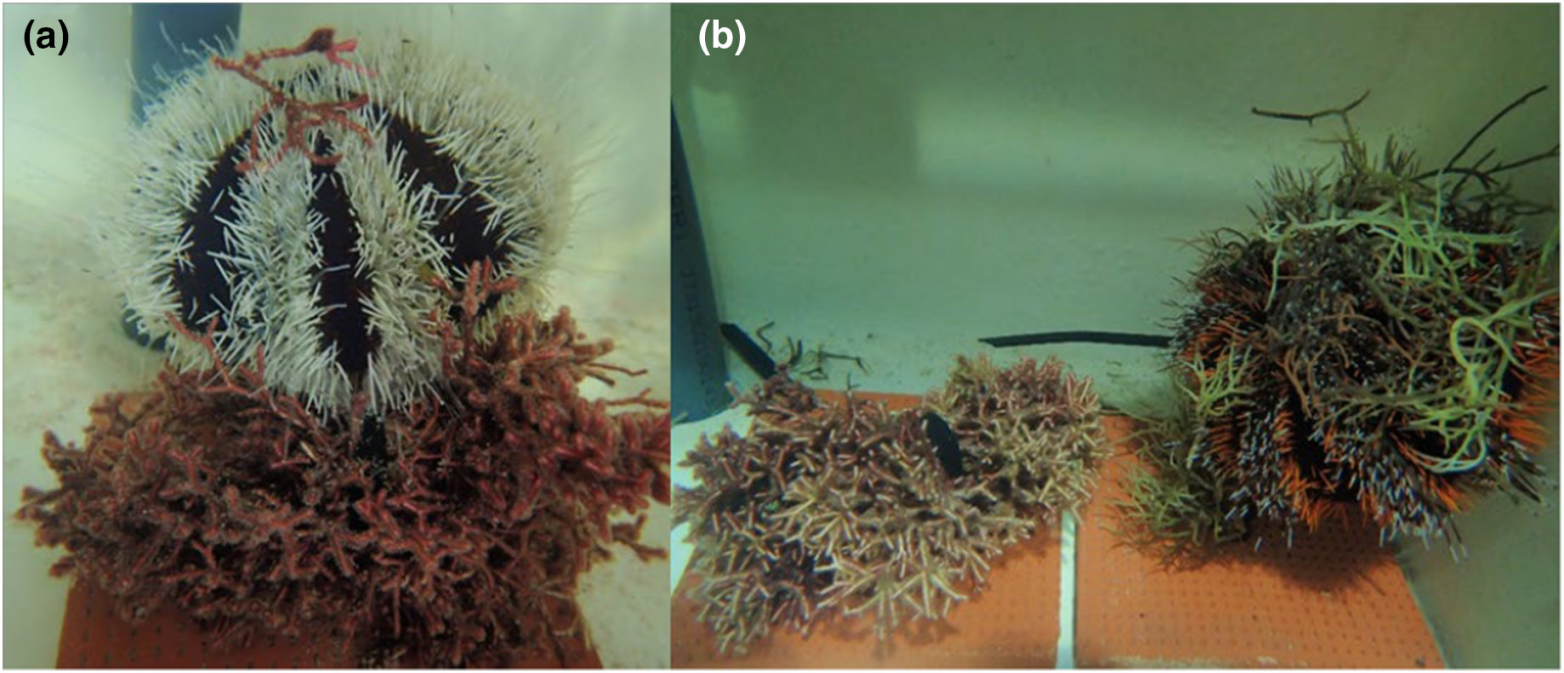
(a) An adult sea urchin *Tripneustes gratilla* feeding on the allelopathic, partially calcified red alga *Galaxaura divaricata* in a “*G*. *divaricata* only”(no‐choice) feeding assay; and (b) *T*. *gratilla* feeding on the nonallelopathic, fleshy, red alga *Gracilaria edulis* in a “*G*. *divaricata* and *G*. *edulis*” (choice) feeding assay. While *T*. *gratilla* fed on both algae in “no‐choice” and “choice” treatments, it consistently consumed less wet‐weight (on average 7 times less) of *G*. *divaricata* than of *G*. *edulis*.

Research permits for feeding experiments were granted by the Dongsha Atoll Marine National Park. We collected *Galaxaura divaricata* and *Gracilaria edulis* (S.G.Gmelin) P.C.Silva from shallow patch reefs and seagrass beds around Dongsha Island. *Gracilaria*, a nonallelopathic, fleshy, red algae genus, is ubiquitous across the Dongsha lagoon and known to be readily consumed by sea urchins and green sea turtles (Hay et al., 1986; Lal Mohan, 1989; Moore et al., 2019; Russell & Balazs, 2015). Algal samples were stored in small recirculating seawater tanks prior to feeding trials, which occurred within 1–12 h after algae collection. Prior to each experimental trial, macroalgae were cleaned to remove any epiphytes and small invertebrates, blotted with a paper towel to remove excess water from the surface, and separated into similar‐sized portions (average wet weight ± SD 38 ± 2.7 g for *G*. *divaricata* and 36 ± 3.3 g for *G*. *edulis*) with their wet weight (Weight_Initial_) measured. For sea urchins, the algae were attached to a ceramic plate that was placed in the middle of the tank (Figure 1). For the turtles the algae portions were given without attachment to ceramic plates as the turtles preferred feeding by lifting the algae off the ground with their mouths.

For sea urchins, algae were offered under three different treatments: “*G*. *edulis* only” (no choice); “*G*. *divaricata* only” (no choice); and “*G*. *edulis* and *G*. *divaricata*” (choice). Food treatments were offered once a day at 5 pm and the algae was left in the tank for 24 h before retrieval. To account for any potential weight changes due to factors other than urchin grazing (e.g., small pieces of algae breaking off due to water flow), a control portion of the respective food treatment was placed in a plastic‐mesh cage and added into each aquarium. The plastic‐mesh cages were used so that controls could not be accessed by the urchins but still be impacted by the water flow in the experimental tank. Treatments were shuffled randomly so that each individual received four experimental trials of each food treatment. After 24 h, all food treatments and controls were retrieved and blotted on tissue paper, and the final wet weight was measured (Weight_End_). Algae consumption by sea urchins (*C*, consumption in grams after 24 h) was estimated using the following equation: *C =* Weight_Initial_ × (control Weight_End_/control Weight_Initial_) − Weight_End_ (Seymour et al., 2013). The percent algae intake (*P*) was calculated as follows: *P =* (*C/*Weight_Inital_) × 100%. The consumption rate (mean ± SD) was calculated for each individual by dividing the consumption (as above) by 24 h. Initial trials revealed that the algae intake proportion of one individual of *E*. *mathaei* was very low. For this reason, the seven individuals of this species were pooled and placed together into one aquarium and the total algae intake proportion was divided by seven.

For green sea turtles, algae treatments were offered once a day at 5 pm and left for 12 h in the tank before retrieval. The sea turtles fed considerably faster than the sea urchins, hence the duration of experimental trials was adjusted to only 12 h, as opposed to the 24‐h trial duration for the sea urchins. On each experimental trial, one of four food treatments was administered: “*G*. *divaricata* only” (no‐choice), *“G*. *edulis* only” (no‐choice), “*G*. *divaricata* and *G*. *edulis*” (choice), and “*G*. *divaricata* mixed with *G*. *edulis*” (mix). Unfortunately, the experiment was cut short as the turtles were scheduled to be released and therefore it was not possible for us to run an equal number of experimental trials for each food treatment. The number of experimental trials per treatment was as follows: four trials for “*G*. *divaricata* only” (no‐choice), one trial for “*G*. *edulis* only” (no‐choice), two trials for “*G*. *divaricata* and *G*. *edulis”* (choice), and three trials for “*G*. *divaricata* mixed with *G*. *edulis*” (mix). The turtles were monitored for the first 15 min of each treatment to ensure they were behaving normally and commenced feeding. We were particularly interested in the turtles' behavioral responses toward the “mix” treatment. This treatment was done to test whether the turtles would continue to avoid feeding on *G*. *divaricata* even if it was more difficult to visually discern when mixed with *G*. *edulis*. After 12 h, the algae samples were retrieved and blotted, and their final wet weight (Weight_End_) was measured. The consumption was estimated by comparing the alga wet weight before and after treatment, *C* = Weight_Initial_ − Weight_End_. The percent algae intake (*P*) was calculated using, *P =* (*C*/Weight_Initial_) × 100%, with *C* being the consumption of algae in grams after 12 h.

### 2.5 Statistical analysis

For the sea urchin data, the macroalgae percent intake (*p*) was logit‐transformed using logit (*p*) = natural log (*p*/(1 − *p*)), where *p* represents the intake proportion linearly scaled to range between 0.005 and 0.995. The logit‐transformed intake proportions were then fitted using a general linear mixed model (GLMM) with the MCMCglmm R package (Hadfield, 2010). We assigned the food type (*G*. *divaricata* or *G*. *edulis*), food treatment (no‐choice or choice), sea urchin species (*D*. *savignyi*, *E*. *calamaris*, or *T*. *gratilla*), and all possible interactions as fixed factors. Data for *D*. *setosum* and *E*. *mathaei* were excluded from the analysis since only one individual of *D*. *setosum*, and the average from seven individuals for *E*. *mathaei* were observed. The following random factors were included in the model: animal ID, the combination of animal ID and food treatment, and the combination of animal ID and experimental trial. Repeated trials were done on the same individuals, and the inclusion of these random factors accounted for repeated measurements and nonindependence of observations. For each model, a total of 200,000 MCMC iterations were performed, including an initial 100,000 burn‐in iterations. Posterior distributions of model parameters were found based on a total of 100,000 MCMC iterations, where every 5th MCMC iteration was selected. Parameter convergence was visually confirmed, and autocorrelation was controlled at *r* < .05. Redundant interaction terms were dropped by using a backward stepwise model selection if the reduced model had a smaller deviance information criterion (DIC). The remaining interaction terms were merged with the respective fixed factors participating in the interaction, and the model was refitted. To conduct multiple comparisons among food treatments, we assessed the disparities in posterior distributions between respective food treatments and sea urchin species.

The same analytical approach was applied to the sea turtle data, where logit‐transformed intake proportions were fitted to a GLMM using the method described above, with food type (*G*. *divaricata* or *G*. *edulis*), food treatment (no‐choice, choice, or mix), and all possible interactions as fixed factors. The following random factors were included in the model: animal ID, the combination of animal ID and food treatment, and the combination of animal ID and experimental trial. To conduct multiple comparisons among food treatments, we assessed the disparities in posterior distributions between respective food treatments and animal IDs.

## 3 RESULTS

### 3.1 Sea urchin feeding assays

**FIGURE 2:**
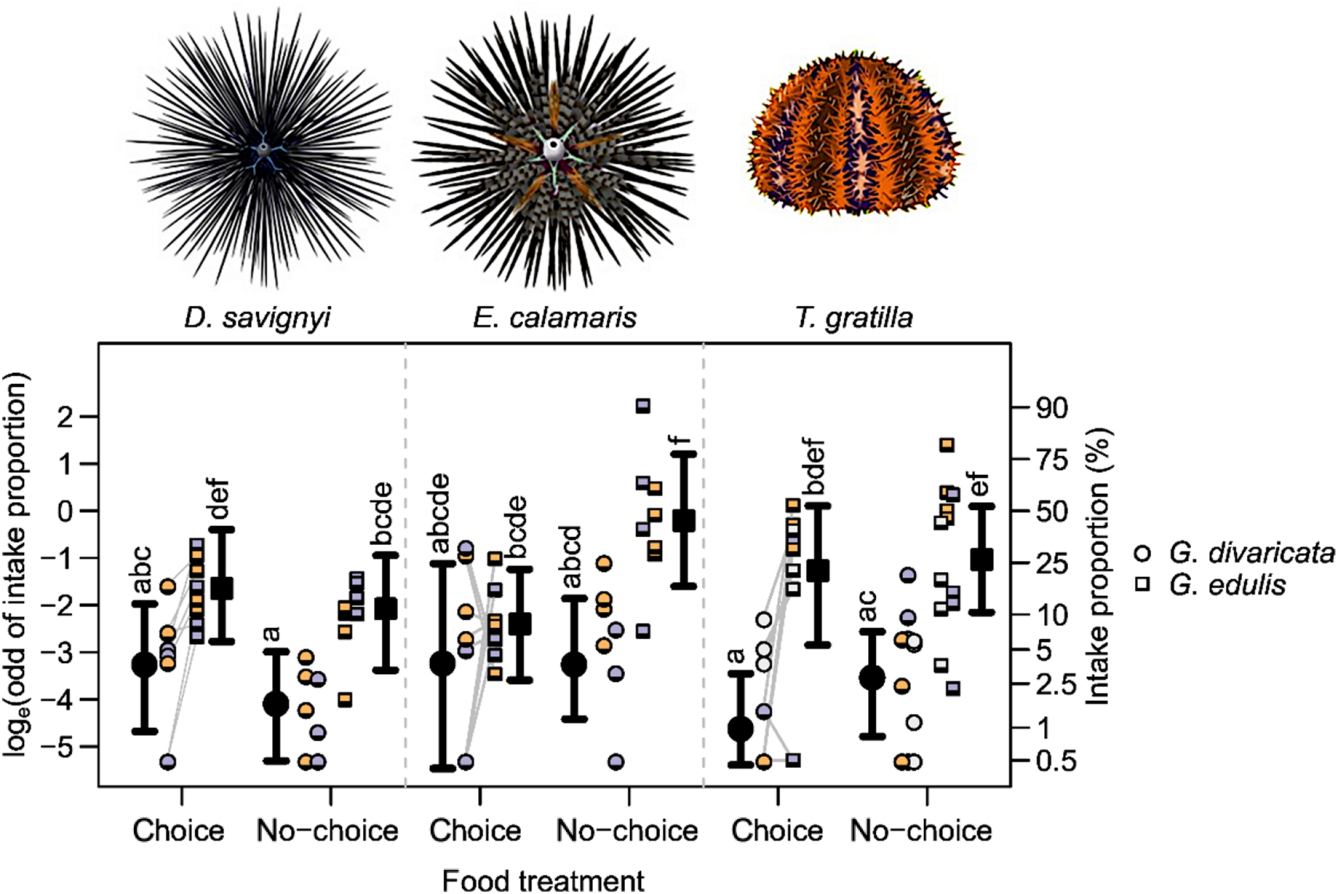
Feeding assays with three species of sea urchins, *Diadema savignyi* (*n* = 2), *Echinothrix calamaris* (*n* = 2), and *Tripneustes gratilla* (*n* = 3) using the allelopathic, partially calcified red alga *Galaxaura divaricata* and the nonallelopathic, fleshy red alga *Gracilaria edulis*. The natural log‐odd of intake proportions (*y*‐axis left) and the percent intake proportions (*y*‐axis right) of *G*. *divaricata* (circles) and *G*. *edulis* (squares) are plotted for each urchin species and for each food treatment: “choice,” where *G*. *divaricata* and *G*. *edulis* were offered at the same time, but as two separate portions; and “no‐choice,” where only one of the two algae species (“*G*. *divaricata* only” or “*G*. *edulis* only”) was offered on a given experimental trial. White‐, orange‐, and purple‐colored symbols represent empirical observations, where each individual is shown in a different color; lines connect symbols from the same experimental trial. Results of the general linear mixed model are shown in black, where black circles/squares and whiskers represent the posterior modes and 95% equal‐tailed credible intervals for each treatment. Lowercase alphabets denote the intake proportion rank based on the results of multiple comparisons; groups that do not share common letters showed no significant difference.

**FIGURE 3:**
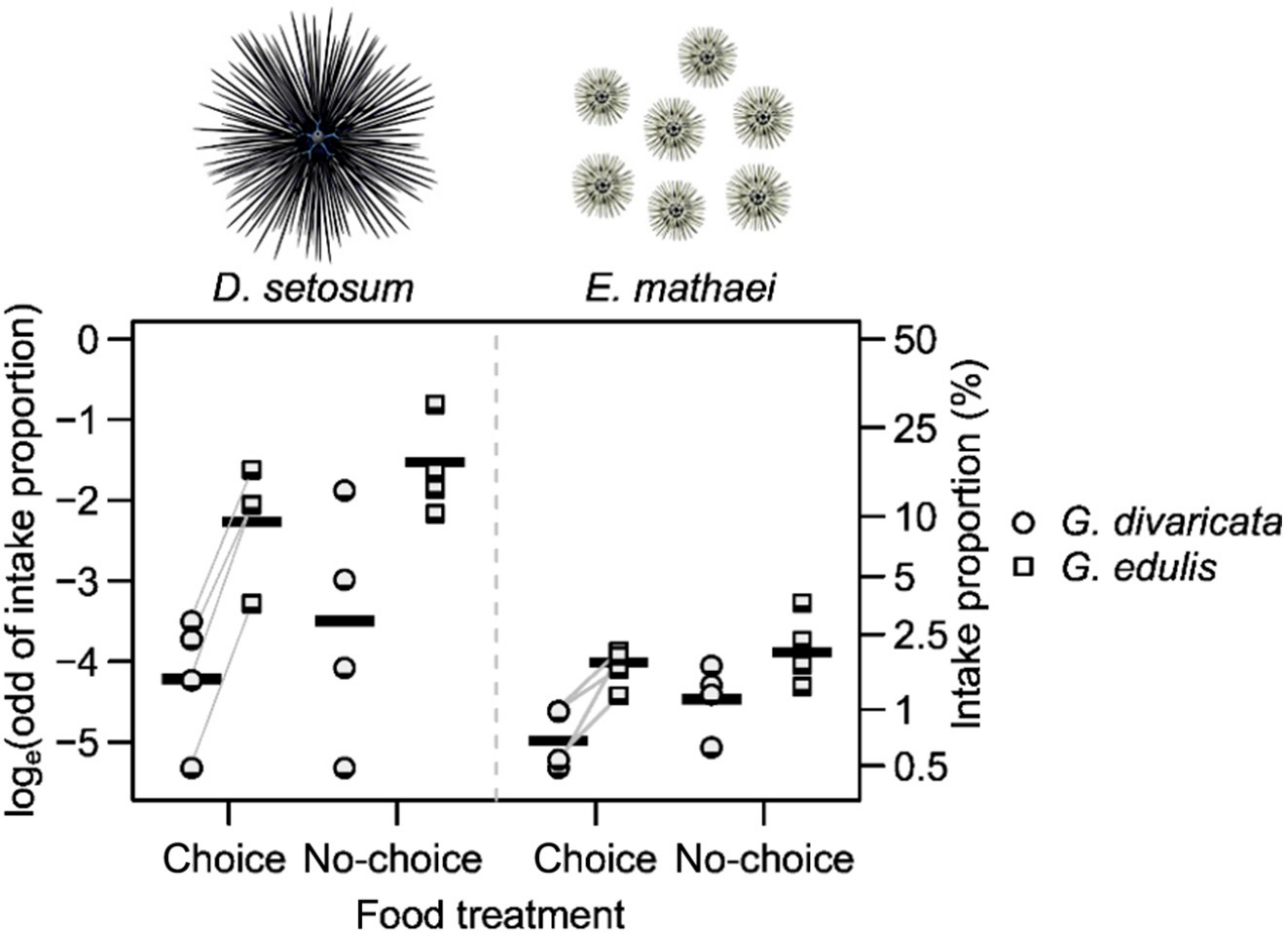
Feeding assays with two species of sea urchins, *Diadema setosum* (*n* = 1), and *Echinometra mathaei* (shown as average for a group of *n* = 7) using the allelopathic, partially calcified red alga *Galaxaura divaricata* and the nonallelopathic, fleshy red alga *Gracilaria edulis*. The natural log‐odd of intake proportions (*y*‐axis left) and the percent intake proportions (*y*‐axis right) of *G*. *divaricata* (circles) and *G*. *edulis* (squares) are plotted for each urchin species and for each food treatment: choice, where *G*. *divaricata* and *G*. *edulis* were offered at the same time, but as two separate portions; and “no‐choice,” where only one of the two algae species (“*G*. *divaricata* only” or “*G*. *edulis* only”) was offered on a given experimental trial. Symbols represent empirical observations; gray lines connect symbols from the same experimental trial. Thick bars indicate the averages of intake proportions. No statistical model was applied due to *n* = 1.

All five species of sea urchins tested in this study consumed both macroalgae, *G*. *divaricata* and *G*. *edulis*. Consumption rate and percent intake were highly correlated (Pearson correlation coefficient > .99). In the no‐choice treatments (“*G*. *divaricata* only” and “*G*. *edulis* only”), feeding rates (g wet weight macroalgae eaten urchin^−1^ 24 h^−1^ ± SD) for *G*. *divaricata* were relatively low across all species, ranging from 0.5 ± 0.3 g urchin^−1^ 24 h^−1^ for *D*. *savignyi*, and 1.91 ± 1.6 g urchin^−1^ 24 h^−1^ for *T*. *gratilla* to 3.1 ± 3 g urchin^−1^ 24 h^−1^ for *E*. *calamaris*. In contrast, the feeding rates of *G*. *edulis* were significantly higher, ranging from 3.9 ± 1.7 g urchin^−1^ 24 h^−1^ for *D*. *savignyi*, and 13.8 ± 9.4 g urchin^−1^ 24 h^−1^ for *T*. *gratilla* to 17.9 ± 1.9 g urchin^−1^ 24 h^−1^ for *E*. *calamaris* (Figures 2 and 3, Table 1). Results of the GLMM indicate that *D*. *savignyi* and *T*. *gratilla* consumed more wet weight of *G*. *edulis* than of *G*. *divaricata* in choice and no‐choice treatments. Interestingly, *E*. *calamaris* consumed significantly more wet weight of *G*. *edulis* than *G*. *divaricata* in the no‐choice treatments but consumed an equal wet weight of both algae in the choice treatments (Figure 2, Tables S1 and S2).

**TABLE 1:** Mean macroalgae consumption rates (grams of macroalgae eaten urchin^−1^ 24 h^−1^ ± SD) for five sea urchin species in no‐choice^a^ feeding assays.

Species	*Galaxaura divaricata*	*Gracilaria edulis*
*Diadema savignyi* (*n* = 2)	0.45 ± 0.29	3.89 ± 1.7
*Diadema setosum* (*n* = 1)	1.66	6.09
*Echinometra mathaei* ^b^	0.27	0.59
*Echinothrix calamaris* (*n* = 2)	3.13 ± 3.03	17.88 ± 1.91
*Tripneustes gratilla* (*n* = 3)	1.91 ± 1.58	13.75 ± 9.39

^a^ No‐choice feeding assay: Only one of the two algae species was offered on a given experimental day.

^b^ The consumption rate was determined for a group of *n* = 7 individuals and divided by 7 to obtain an estimate of the consumption rate per urchin.

### 3.2 Green sea turtle feeding assays

**FIGURE 4:**
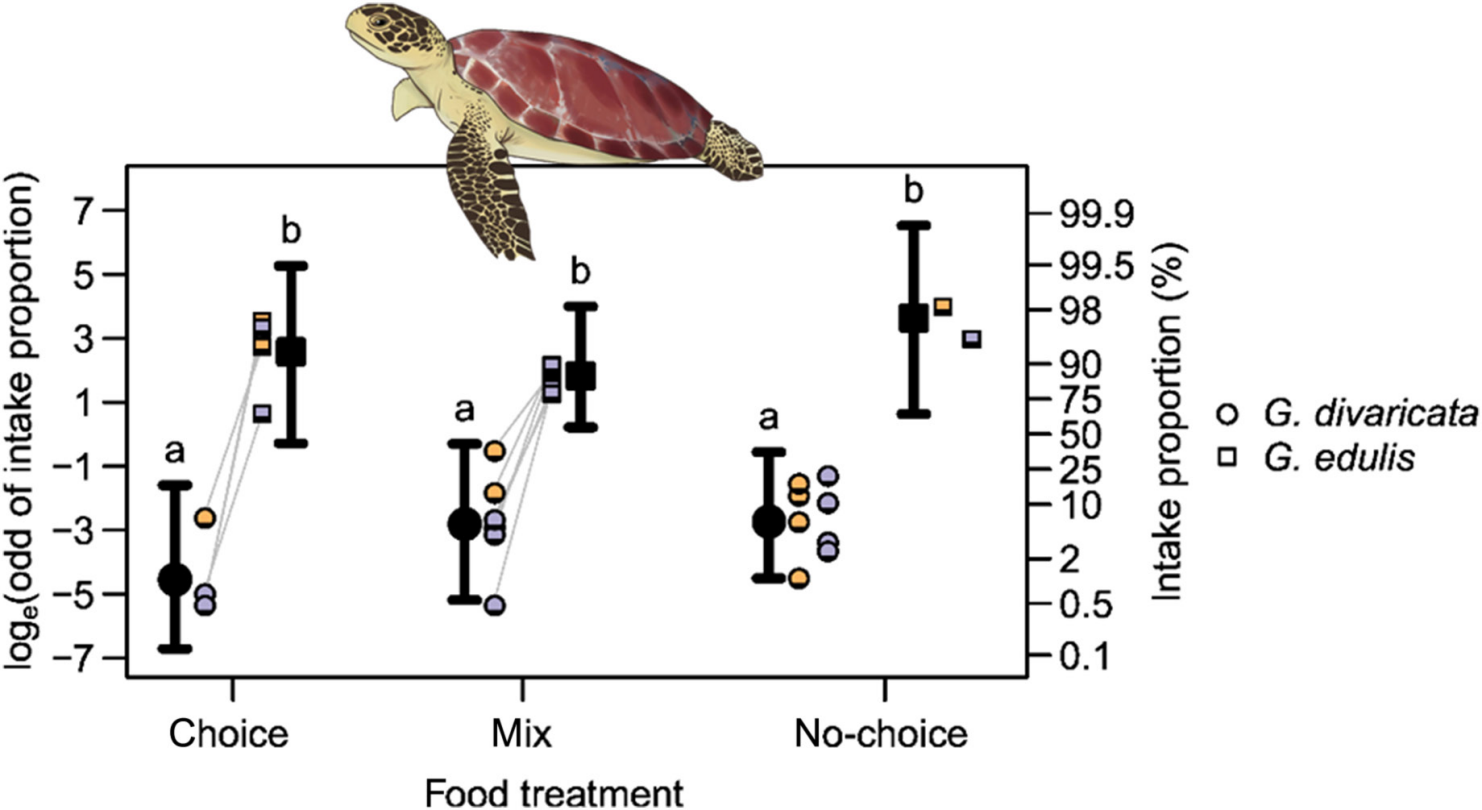
Feeding assays with two green sea turtles (*Chelonia mydas*) using the allelopathic, partially calcified red alga *Galaxaura divaricata* and the nonallelopathic, fleshy red alga *Gracilaria edulis*. The natural log‐odd of intake proportions (*y*‐axis left) and the percent intake proportions (*y*‐axis right) of *G*. *divaricata* (circles) and *G*. *edulis* (squares) are plotted for each food treatment: “choice,” where *G*. *divaricata* and *G*. *edulis* were offered at the same time, but as two separate portions; “mix,” where *G*. *divaricata* and *G*. *edulis* were mixed and offered as one portion; and “no‐choice,” where only one of the two algae species (“*G*. *divaricata* only” or “*G*. *edulis* only”) was offered on a given experimental trial. Orange‐ and purple‐colored symbols represent empirical observations, where each individual is shown in a different color; lines connect symbols from the same experimental trial. Results of the general linear mixed model are shown in black, where black circles/squares and whiskers represent the posterior modes and 95% equal‐tailed credible intervals for each treatment. Lowercase alphabets denote the intake proportion rank based on the results of multiple comparisons; groups that do not share common letters showed no significant difference.

Both turtles preferred feeding on *G*. *edulis*. About 90% of *G*. *edulis* was consumed within the first 15–20 min of the trials for all treatment types (no‐choice, choice, and mix). In contrast, both turtles strictly avoided feeding on *G*. *divaricata* (Video S1) for all treatment types. When offered the “mix” treatment the turtles continued to selectively feed on *G*. *edulis*. The turtles cherry‐picked pieces of *G*. *edulis* out of the mix and, if *G*. *divaricata* was picked by accident, they would spit it back out. Sometimes, the turtles picked the mixed algae portion up with their mouths and shook it to separate the two algae species. They then continued to feed on *G*. *edulis* only, avoiding *G*. *divaricata* until there was only *G*. *divaricata* left in the tank (Video S2). The results of the GLMM showed that the intake proportion or wet weight change (initial versus end) of *G*. *edulis* (range 90%–98%) was significantly higher than that of *G*. *divaricata* (range 1%–8%) for all treatments (no‐choice, choice, and mix), and did not significantly differ among treatments (Figure 4, Table S1 and S3). There was a minimal weight change of *G*. *divaricata* of 5.02 g for “no‐choice” treatments, 1.11 g for the “choice” treatments, and 5.43 g for the “mix” treatments. However, this was most likely due to the stronger water flow in the large tanks housing the turtles compared to the smaller tanks used for the urchins, the difficulty in collecting smaller algae pieces that had broken off in the larger tanks, and the lack of algae weight change controls used in the sea turtle trials, rather than to actual consumption by the turtles.

## 4 DISCUSSION

Allelopathic macroalgae can pose a threat to the health of coral reefs, so understanding whether herbivores will consume or avoid such algae can provide insights as to whether the abundance of allelopathic algae could be regulated via top‐down control. *Galaxaura divaricata* is known to proliferate on degraded coral reefs, potentially causing long‐lasting overgrowths, and limiting coral recovery. Here we tested the feeding preference of five species of sea urchins and two green sea turtles (*Chelonia mydas*) for the allelopathic, partially calcified red alga *G*. *divaricata* compared with the nonallelopathic, fleshy *Gracilaria edulis*. Overall, we found that *G*. *divaricata* was not preferred by the herbivores tested, but that urchins did consume the allelopathic alga to an extent.

First, our results showed that the five sea urchin species tested feed on both *G*. *divaricata* and *G*. *edulis*. In no‐choice treatments, which involved the presentation of either “*G*. *divaricata* only” or “*G*. *edulis* only” on a given day, the urchins consistently ingested less (wet weight) of *G*. *divaricata* than of *G*. *edulis*. The overall lower wet weight of *G*. *divaricata* consumed by urchins in this study compared to *G*. *edulis* is consistent with previous studies showing that calcified macroalgae that are also rich in bioactive compounds are the least preferred by sea urchins, compared to fleshy, soft, and filamentous macroalgae (Coppard & Campbell, 2007). In “choice” treatments, which involved the presentation of both *G*. *divaricata* and *G*. *edulis* at the same time, the urchin species *Diadema savignyi*, *Diadema setosum*, and *Tripneustes gratilla* preferred *G*. *edulis* over *G*. *divaricata*, while *Echinothrix calamaris* and *Echinometra mathaei* consumed approximately equal wet weights of both algae. *Echinothrix calamaris* consumed the highest average wet weight of *G*. *divaricata* of all five urchin species tested [“*G*. *divaricata* only” (no‐choice) 3.1 ± 3 g urchin^−1^ 24 h^−1^], which may be due to the much larger body size of *E*. *calamaris* (Coppard & Campbell, 2007; Klumpp et al., 1993). However, the consumed wet weight of *G*. *divaricata* by *E*. *calamaris* was considerably lower than the average wet weight intake of [“*G*. *edulis* only” (no‐choice) 17.9 ± 1.9 g urchin^−1^ 24 h^−1^]. The consumption of *G*. *divaricata* in both choice and no‐choice treatments by some urchin species indicates that while it may not be their favored algae, some urchins may be consumers of *G*. *divaricata* if it is present in their natural habitat.

While sea urchins are generalist grazers and require a mixed diet for optimal growth, they are known to show preferences for particular algal species (Coppard & Campbell, 2007). For instance, *T*. *gratilla* generally exhibits a low preference for calcified algae (Klumpp et al., 1993; Seymour et al., 2013), which is supported by the present study, where *T*. *gratilla* consistently preferred feeding on the fleshy *G*. *edulis* over the partially calcified *G*. *divaricata*. Urchin feeding preference depends on the species and may vary among species of the same genus (Coppard & Campbell, 2007; Lawrence & Sammarco, 1982). For instance, *Diadema antillarum*, an urchin species native to the Caribbean consumed considerably higher quantities of *Galaxaura* sp. (14.8 g urchin^−1^ 24 h^−1^) in similar no‐choice feeding experiments (Solandt & Campbell, 2001). In contrast, the Pacific *Diadema* species tested here, namely *D*. *setosum* and *D*. *savignyi* consumed very little *G*. *divaricata* (range 0.5–1.7 g urchin^−1^ 24 h^−1^) in no‐choice treatments. Our results lend support to the findings of previous studies which have shown that, unlike *D*. *antillarum*, the Pacific *Diadama* species, *D*. *setosum* and *D*. *savignyi* are not known to exert a major top‐down control on macroalgae, and rather are associated with the erosion of coral in the Indo Pacific and the Red Sea (Cheal et al., 2010; Goh & Lim, 2015; Lawrence, 2013; Mokady et al., 1996).

Second, observations from this study suggest that green sea turtles, *Chelonia mydas* strictly avoid feeding on the chemically rich and partially calcified *G*. *divaricata* and only feed on the nonallelopathic, fleshy *Gracilaria edulis*. These observations agree with previous studies that looked at dietary choices and stomach contents in *C*. *mydas*, showing that green sea turtles strongly prefer seagrass and fleshy or filamentous macroalgae, similar to *G*. *edulis*, but do not consume calcified macroalgae (Amorocho & Reina, 2007; Arthur et al., 2009; Arthur & Balazs, 2008; Carrión‐Cortez et al., 2010; Garnett et al., 1985; Hendrickson, 1958; Ross, 1985; Russell & Balazs, 2009).

Herbivorous fishes are also major consumers of macroalgae; however, few studies that have investigated herbivorous fish feeding interactions with *Galaxaura* suggest most species avoid feeding on *Galaxaura* (Loffler et al., 2015; Mantyka & Bellwood, 2007a, 2007b; Nieder et al., 2022). Damselfishes and young parrotfishes are known to feed on macro‐ and micro‐epiphytes from the surface of *G*. *divaricata*, but not on *G*. *divaricata* itself (Nieder et al., 2022). According to our preliminary observations made in Dongsha Atoll, only two species of rabbitfishes, *Siganus guttatus* and *S*. *spinus* were witnessed occasionally grazing on *G*. *divaricata*.

Several factors (e.g., nutritional, structural, chemical) may contribute to why *Galaxaura* is not readily consumed by herbivores (Cox & Murray, 2006; Hay, 2009). One hypothesis is that *Galaxaura* may contain fewer nutrients and be lower in organic contents compared to fleshy macroalgae (Rotjan & Lewis, 2005), due to its relatively high calcium carbonate content (~70%) (Solandt & Campbell, 2001). However, a recent study on coralline algae showed that, when compared on a per‐volume basis, heavily calcified coralline algae and uncalcified fleshy algae have similar proportions of organic tissue (Haberman & Martone, 2023). Haberman and Martone (2023) argued that the widespread observations that herbivores prefer uncalcified‐fleshy algae over calcified algae cannot be explained by differences in caloric content, indicating that another mechanism must account for the avoidance of calcified coralline algae by many herbivores (Haberman & Martone, 2023). *G*. *divaricata* is partially calcified, only depositing calcium carbonate into the cell walls of its outer thalli. The inner noncalcified medulla contains soft, fleshy cell layers of organic tissue (Liu et al., 2013) that could potentially serve as food, containing caloric and nutritional value to herbivores. Others have argued that calcified algae may be more energetically costly for fish and sea urchins to digest (Duffy & Hay, 1990, 1994). Fleshy macroalgae may require less energy for digestion, providing more cost‐effective access to nutrients (Steneck, 1988), and consequently are a preferred food source for a wide range of herbivorous species (Seymour et al., 2013). More research into the energetic and nutritional value of *G*. *divaricata* tissues (e.g., protein, soluble carbohydrate, fiber content, and nitrogen storage) is needed to better evaluate the nutritional status of *G*. *divaricata*.

Another hypothesis that could explain the observed avoidance or lack of effective removal of *Galaxaura* by herbivores may be attributed to the alga's arsenal of secondary chemicals (Anulika et al., 2016; Hay & Fenical, 1988; James Graham et al., 2009). These chemicals may be experienced as an aversive “smell” or “taste” by some herbivores or may even result in digestive or other health issues. *Galaxaura* produces a variety of secondary metabolites including terpenes, aromatic compounds, acetogenins, amino acid‐derived substances, and polyphenols (Abdel‐Raouf et al., 2017; Al‐Enazi et al., 2018; Hay, 1986, 1996; Rasher et al., 2011). Some of which are also produced by some terrestrial plants and are known for their herbivore deterrence effect (Hay & Fenical, 1988; Paul et al., 2001). According to a field study, a diet composed of only *Galaxaura* had a detrimental effect on *D*. *antillarum*, resulting in spine loss and shrinking gonads (Sammarco, 1982). However, our study shows that urchins do consume *Galaxaura*, but only in smaller quantities. It is hypothesized that aquatic animals may incorporate small amounts of macroalgae with high levels of bioactive compounds into their diets to benefit from potential prophylactic effects, boost immunocompetence, or treat pathogens or parasites (Vaughan et al., 2023). Some of the many secondary metabolites in *Galaxaura* may have antifungal or anti‐pathogenic effects (Abdel‐Raouf et al., 2017; Al‐Enazi et al., 2018), and sea urchins may benefit from increased physical resilience to pathogens via feeding on small quantities of *Galaxaura*.

A caveat of this study is that the data presented here reflect the behavioral choices of animals in captivity and whether these observations can be directly transferable in the wild should be treated with caution. While feeding behaviors observed in captivity can be indicative of how animals could behave in the wild, the behaviors of animals are complex and may vary with a range of factors (e.g., age, physical condition, season, food availability, environmental conditions) (Prior et al., 2016; Souza et al., 2008; Stimson et al., 2007; Vadas, 1977). Therefore, follow‐up feeding studies in the wild are necessary to confirm the results of experiments conducted in captivity. In addition, this study is limited by the low number of individual green sea turtles and sea urchins available for trials. The low number of sea urchins per species was the result of the shear paucity of sea urchins encountered across the Dongsha lagoon [diurnal densities <1 individual per 100 m^2^ (Nieder et al., 2019)]. Therefore, our data may not represent the “true” algae consumption rates for the species examined but may be a valuable ballpark estimate to guide future studies.

## 5 CONCLUSION

Understanding the feeding preferences of coral reef macroherbivores, such as sea urchins and green sea turtles, is crucial for devising effective strategies to mitigate the overgrowth of coral competitive and allelopathic macroalgae, like *Galaxaura*. Our data suggests that sea urchins and green sea turtles may not act as major controlling agents of *G*. *divaricata* abundance. As an alternative strategy, effective large‐scale removal of *G*. *divaricata* may only be achieved through human intervention. Further investigation is needed to determine the impact of sea urchin in situ grazing on the abundance of *G*. *divaricata* in the wild. Such studies will enhance our understanding of how different marine herbivores contribute to the regulation of overgrowths of coral competitive and allelopathic macroalgae on coral reefs, assisting in management decisions for reefs facing increasing and multifaceted stressors.

## AUTHOR CONTRIBUTIONS

**Carolin Nieder:** Conceptualization (equal); data curation (equal); formal analysis (equal); investigation (equal); methodology (equal); software (equal); visualization (equal); writing – original draft (equal); writing – review and editing (equal). **Siobhan Jean Heatwole:** Investigation (equal); writing – review and editing (equal). **Chen‐Pan Liao:** Data curation (equal); formal analysis (equal); methodology (equal); software (equal); validation (equal); visualization (equal); writing – review and editing (equal). **Chen‐Lu Lee:** Formal analysis (equal); validation (equal); visualization (equal). **Chaolun Allen Chen:** Investigation (equal); writing – review and editing (equal). **Shao‐Lun Liu:** Conceptualization (equal); funding acquisition (equal); investigation (equal); project administration (equal); resources (equal); supervision (equal); validation (equal); visualization (equal); writing – review and editing (equal).

## CONFLICT OF INTEREST STATEMENT

The authors have declared that no competing interests exist.

### OPEN RESEARCH BADGES

This article has earned Open Data, Open Materials and Preregistered Research Design badges. Data, materials and the preregistered design and analysis plan are available at https://doi.org/10.6084/m9.figshare.25357198.v2.

## Supporting information


Video S1.

Video S2.

Appendix S1.

## Data Availability

The authors confirm that the data supporting the findings of this study are available within the article and its Supporting Information. R codes and data for statistical analyses and figures can be accessed online via the figshare website at the following link: https://doi.org/10.6084/m9.figshare.25357198.v2.
