# Supplementary material for: Testing sea urchin and green sea turtle consumption of the allelopathic macroalga Galaxaura divaricata
Source: Ecol Evol. 2024 Apr 25;14(4):e11324. doi: 10.1002/ece3.11324 (PMC11045560; doi:10.1002/ece3.11324)
Supplement: Supplementary file 3 — Appendix S1. [file ECE3-14-e11324-s002.docx]

## 10.Appendix

**Table S1.** Posterior distributions of intake proportion of algae consumed by three sea urchin species including, **(a)** *Diadema savignyi*, **(b)** *Echinothrix calamaris*, **(c)** *Tripneustes gratilla*, as well as the **(d)** green sea turtle, *Chelonia mydas*. Empirical averages for the intake proportions of sea urchins **(e)** *D. setosum*, and **(f)** *E. mathaei* are provided here since those data were not fitted by a GLMM. Abbreviations: *N*, number of individuals; CIs, equal-tailed credible intervals.

| **Species** | | **Food treatment** | | **Food type** | | ***N*** | | **Posterior mode** | | **95% CIs** |
| --- | --- | --- | --- | --- | --- | --- | --- | --- | --- | --- |
| **(a)** | *D. savignyi* | | No-choice | | *Galaxaura* | | 2 | | 0.0128 | (0.0051, 0.0484) |
|  | *D. savignyi* | | No-choice | | *Gracilaria* | | 2 | | 0.0876 | (0.0318, 0.2654) |
|  | *D. savignyi* | | Choice | | *Galaxaura* | | 2 | | 0.0367 | (0.0087, 0.1308) |
|  | *D. savignyi* | | Choice | | *Gracilaria* | | 2 | | 0.1680 | (0.0528, 0.3630) |
| **(b)** | *E. calamaris* | | No-choice | | *Galaxaura* | | 2 | | 0.0479 | (0.0122, 0.1489) |
|  | *E. calamaris* | | No-choice | | *Gracilaria* | | 2 | | 0.4408 | (0.1658, 0.7675) |
|  | *E. calamaris* | | Choice | | *Galaxaura* | | 2 | | 0.0393 | (0.0046, 0.2748) |
|  | *E. calamaris* | | Choice | | *Gracilaria* | | 2 | | 0.0804 | (0.0246, 0.2282) |
| **(c)** | *T. gratilla* | | No-choice | | *Galaxaura* | | 3 | | 0.0250 | (0.0085, 0.0754) |
|  | *T. gratilla* | | No-choice | | *Gracilaria* | | 3 | | 0.2598 | (0.0980, 0.5068) |
|  | *T. gratilla* | | Choice | | *Galaxaura* | | 3 | | 0.0106 | (0.0040, 0.0292) |
|  | *T. gratilla* | | Choice | | *Gracilaria* | | 3 | | 0.2243 | (0.0502 ,0.5098) |
| **(d)** | *C. mydas* | | No-choice | | *Galaxaura* | | 3 | | 0.0706 | (0.0121, 0.3466) |
|  | *C. mydas* | | No-choice | | *Gracilaria* | | 3 | | 0.9714 | (0.6640, 0.9983) |
|  | *C. mydas* | | Mixed | | *Galaxaura* | | 3 | | 0.0627 | (0.0084, 0.4146) |
|  | *C. mydas* | | Mixed | | *Gracilaria* | | 3 | | 0.8471 | (0.5202, 0.9730) |
|  | *C. mydas* | | Choice | | *Galaxaura* | | 3 | | 0.0113 | (0.0016, 0.1839) |
|  | *C. mydas* | | Choice | | *Gracilaria* | | 2 | | 0.9264 | (0.4325, 0.9923) |
| **Species** | | **Food treatment** | | **Food type** | | ***N*** | | **Empirical average** | |  |
| **(e)** | *D. setosum* | Choice | | *Galaxaura* | | 1 | | 0.0152 | |  |
|  | *D. setosum* | Choice | | *Gracilaria* | | 1 | | 0.0951 | |  |
|  | *D. setosum* | No-choice | | *Galaxaura* | | 1 | | 0.0282 | |  |
|  | *D. setosum* | No-choice | | *Gracilaria* | | 1 | | 0.1648 | |  |
| **(f)** | *E. mathaei* | Choice | | *Galaxaura* | | 1 | | 0.0073 | |  |
|  | *E. mathaei* | Choice | | *Gracilaria* | | 1 | | 0.0166 | |  |
|  | *E. mathaei* | No-choice | | *Galaxaura* | | 1 | | 0.0118 | |  |
|  | *E. mathaei* | No-choice | | *Gracilaria* | | 1 | | 0.0210 | |  |

**Table S2.** Results of multiple comparisons of algae consumption odds ratio (OR) among three sea urchin species, *Diadema savignyi*, *Echinothrix calamaris* and *Tripneustes gratilla*. Abbreviations: GD, *Galaxaura divaricata*; GE, *Gracilaria edulis*; CIs, equal-tailed credible intervals.

| **Comparison** | **Posterior mode** | **95% CIs** | ***P*_MCMC_** |
| --- | --- | --- | --- |
| *D. savignyi*, no-choice GD vs *D. savignyi*, no-choice GE | 0.1216 | (0.0302, 0.6116) | 0.0173 |
| *D. savignyi*, no-choice GD vs *D. savignyi*, choice GD | 0.4708 | (0.0777, 2.2716) | 0.2665 |
| *D. savignyi*, no-choice GD vs *D. savignyi*, choice GE | 0.0792 | (0.0195, 0.3761) | 0.0041 |
| *D. savignyi*, no-choice GD vs *E. calamaris*, no-choice GD | 0.3192 | (0.0586, 1.8264) | 0.1767 |
| *D. savignyi*, no-choice GD vs *E. calamaris*, no-choice GE | 0.0167 | (0.0031, 0.1128) | 0.0008 |
| *D. savignyi*, no-choice GD vs *E. calamaris*, choice GD | 0.4443 | (0.0341, 4.5104) | 0.3952 |
| *D. savignyi*, no-choice GD vs *E. calamaris*, choice GE | 0.1501 | (0.0356, 0.9715) | 0.0449 |
| *D. savignyi*, no-choice GD vs T. gratilla, no-choice GD | 0.4990 | (0.1222, 3.0350) | 0.4746 |
| *D. savignyi*, no-choice GD vs T. gratilla, no-choice GE | 0.0383 | (0.0093, 0.2254) | 0.0015 |
| *D. savignyi*, no-choice GD vs T. gratilla, choice GD | 1.2575 | (0.3079, 6.4231) | 0.6757 |
| *D. savignyi*, no-choice GD vs T. gratilla, choice GE | 0.0628 | (0.0104, 0.4468) | 0.0087 |
| *D. savignyi*, no-choice GE vs *D. savignyi*, choice GD | 2.3160 | (0.5151, 15.7249) | 0.1935 |
| *D. savignyi*, no-choice GE vs *D. savignyi*, choice GE | 0.5991 | (0.1344, 2.6905) | 0.4581 |
| *D. savignyi*, no-choice GE vs *E. calamaris*, no-choice GD | 2.5552 | (0.4065, 13.8543) | 0.3188 |
| *D. savignyi*, no-choice GE vs *E. calamaris*, no-choice GE | 0.1658 | (0.0212, 0.8086) | 0.0377 |
| *D. savignyi*, no-choice GE vs *E. calamaris*, choice GD | 2.5283 | (0.2173, 31.7977) | 0.4090 |
| *D. savignyi*, no-choice GE vs *E. calamaris*, choice GE | 1.0416 | (0.2442, 7.3072) | 0.7798 |
| *D. savignyi*, no-choice GE vs *T. gratilla*, no-choice GD | 3.5853 | (0.8470, 22.6184) | 0.0778 |
| *D. savignyi*, no-choice GE vs *T. gratilla*, no-choice GE | 0.3192 | (0.0618, 1.6365) | 0.1515 |
| *D. savignyi*, no-choice GE vs *T. gratilla*, choice GD | 8.6108 | (2.1004, 46.5649) | 0.0074 |
| *D. savignyi*, no-choice GE vs *T. gratilla*, choice GE | 0.5181 | (0.0711, 3.2470) | 0.4119 |
| *D. savignyi*, choice GD vs *D. savignyi*, choice GE | 0.1978 | (0.0465, 0.8602) | 0.0354 |
| *D. savignyi*, choice GD vs *E. calamaris*, no-choice GD | 0.7033 | (0.1140, 5.2224) | 0.7847 |
| *D. savignyi*, choice GD vs *E. calamaris*, no-choice GE | 0.0454 | (0.0061, 0.3192) | 0.0064 |
| *D. savignyi*, choice GD vs *E. calamaris*, choice GD | 0.9515 | (0.0654, 12.0651) | 0.9349 |
| *D. savignyi*, choice GD vs *E. calamaris*, choice GE | 0.3565 | (0.0641, 2.6044) | 0.3304 |
| *D. savignyi*, choice GD vs *T. gratilla*, no-choice GD | 1.5361 | (0.2362, 8.7515) | 0.6742 |
| *D. savignyi*, choice GD vs *T. gratilla*, no-choice GE | 0.1170 | (0.0180, 0.6611) | 0.0211 |
| *D. savignyi*, choice GD vs *T. gratilla*, choice GD | 3.2471 | (0.6266, 20.6573) | 0.1572 |
| *D. savignyi*, choice GD vs *T. gratilla*, choice GE | 0.1443 | (0.0214, 1.2984) | 0.0778 |
| *D. savignyi*, choice GE vs *E. calamaris*, no-choice GD | 4.1717 | (0.7100, 23.5129) | 0.1169 |
| *D. savignyi*, choice GE vs *E. calamaris*, no-choice GE | 0.2178 | (0.0382, 1.4134) | 0.1011 |
| *D. savignyi*, choice GE vs *E. calamaris*, choice GD | 3.9304 | (0.3921, 56.4617) | 0.2098 |
| *D. savignyi*, choice GE vs *E. calamaris*, choice GE | 2.3014 | (0.3877, 11.2038) | 0.3474 |
| *D. savignyi*, choice GE vs *T. gratilla*, no-choice GD | 7.6433 | (1.3561, 34.5071) | 0.0224 |
| *D. savignyi*, choice GE vs *T. gratilla*, no-choice GE | 0.5675 | (0.1075, 2.6530) | 0.4115 |
| *D. savignyi*, choice GE vs *T. gratilla*, choice GD | 14.7501 | (3.4663, 75.0967) | 0.0024 |
| *D. savignyi*, choice GE vs *T. gratilla*, choice GE | 0.9352 | (0.1150, 5.0968) | 0.7875 |
| *E. calamaris*, no-choice GD vs *E. calamaris*, no-choice GE | 0.0508 | (0.0099, 0.3684) | 0.0048 |
| *E. calamaris*, no-choice GD vs *E. calamaris*, choice GD | 1.1459 | (0.0936, 12.2755) | 0.9016 |
| *E. calamaris*, no-choice GD vs *E. calamaris*, choice GE | 0.4079 | (0.1032, 2.8638) | 0.4277 |
| *E. calamaris*, no-choice GD vs *T. gratilla*, no-choice GD | 2.1594 | (0.3069, 9.5978) | 0.4643 |
| *E. calamaris*, no-choice GD vs *T. gratilla*, no-choice GE | 0.1339 | (0.0247, 0.7539) | 0.0284 |
| *E. calamaris*, no-choice GD vs *T. gratilla*, choice GD | 4.2733 | (0.8464, 22.6290) | 0.0795 |
| *E. calamaris*, no-choice GD vs *T. gratilla*, choice GE | 0.2317 | (0.0294, 1.4935) | 0.1049 |
| *E. calamaris*, no-choice GE vs *E. calamaris*, choice GD | 14.9030 | (1.6693, 258.4436) | 0.0262 |
| *E. calamaris*, no-choice GE vs *E. calamaris*, choice GE | 8.3825 | (1.5304, 49.6335) | 0.0183 |
| *E. calamaris*, no-choice GE vs *T. gratilla*, no-choice GD | 29.9296 | (5.0474, 189.0431) | 0.0017 |
| *E. calamaris*, no-choice GE vs *T. gratilla*, no-choice GE | 2.9267 | (0.3851, 14.3476) | 0.3184 |
| *E. calamaris*, no-choice GE vs *T. gratilla*, choice GD | 74.5072 | (12.4559, 388.4457) | 0.0004 |
| *E. calamaris*, no-choice GE vs *T. gratilla*, choice GE | 3.6183 | (0.4568, 26.2038) | 0.2130 |
| *E. calamaris*, choice GD vs *E. calamaris*, choice GE | 0.5563 | (0.0478, 4.5067) | 0.4711 |
| *E. calamaris*, choice GD vs *T. gratilla*, no-choice GD | 1.6673 | (0.1410, 18.5335) | 0.6844 |
| *E. calamaris*, choice GD vs *T. gratilla*, no-choice GE | 0.1172 | (0.0098, 1.3558) | 0.0831 |
| *E. calamaris*, choice GD vs *T. gratilla*, choice GD | 3.7698 | (0.3249, 40.5543) | 0.2570 |
| *E. calamaris*, choice GD vs *T. gratilla*, choice GE | 0.1459 | (0.0143, 2.7046) | 0.1850 |
| *E. calamaris*, choice GE vs *T. gratilla*, no-choice GD | 3.5347 | (0.6363, 17.4968) | 0.1363 |
| *E. calamaris*, choice GE vs *T. gratilla*, no-choice GE | 0.2669 | (0.0484, 1.2950) | 0.0951 |
| *E. calamaris*, choice GE vs *T. gratilla*, choice GD | 7.1742 | (1.5606, 37.3844) | 0.0163 |
| *E. calamaris*, choice GE vs *T. gratilla*, choice GE | 0.3963 | (0.0559, 2.5859) | 0.2953 |
| *T. gratilla*, no-choice GD vs *T. gratilla*, no-choice GE | 0.0726 | (0.0177, 0.3416) | 0.0017 |
| *T. gratilla*, no-choice GD vs *T. gratilla*, choice GD | 2.2631 | (0.5787, 9.2554) | 0.2175 |
| *T. gratilla*, no-choice GD vs *T. gratilla*, choice GE | 0.1290 | (0.0189, 0.6514) | 0.0186 |
| *T. gratilla*, no-choice GE vs *T. gratilla*, choice GD | 34.5940 | (7.2903, 119.2839) | 0.0002 |
| *T. gratilla*, no-choice GE vs *T. gratilla*, choice GE | 1.8589 | (0.2535, 8.5984) | 0.6485 |
| *T. gratilla*, choice GD vs *T. gratilla*, choice GE | 0.0490 | (0.0099, 0.2189) | 0.0009 |


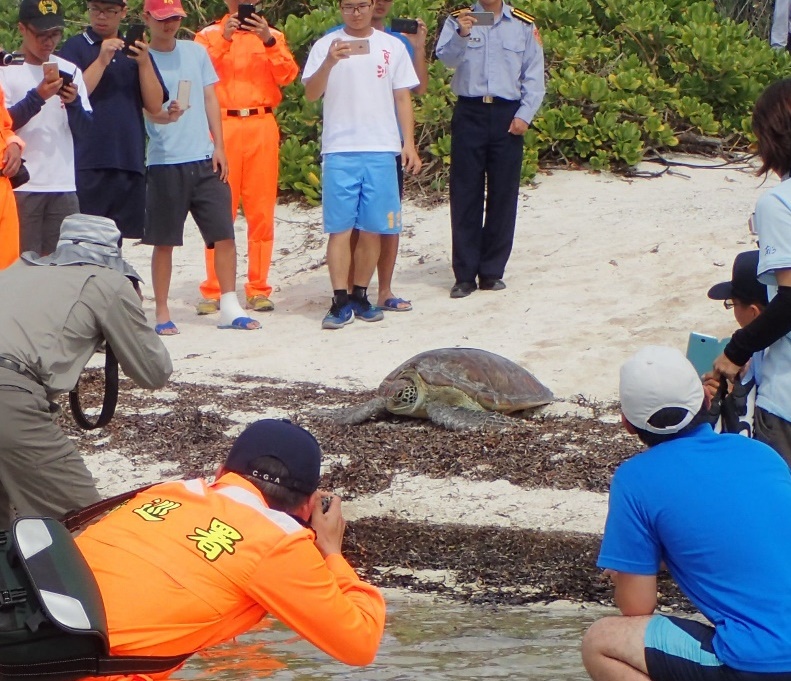


**Figure S1.** Release of a female *Chelonia mydas* used for feeding assays in this study. The individual was rescued from illegal fishermen by the Taiwanese coast guards and brought to the Dongsha Atoll National Park for rehabilitation. Illegal capture of sea turtles is a common practice around Dongsha and is apparently a large threat to the local nesting population. For this to be avoided and for conservation measures to be effective, adequate law enforcement must occur within and around the Dongsha Atoll National Park.


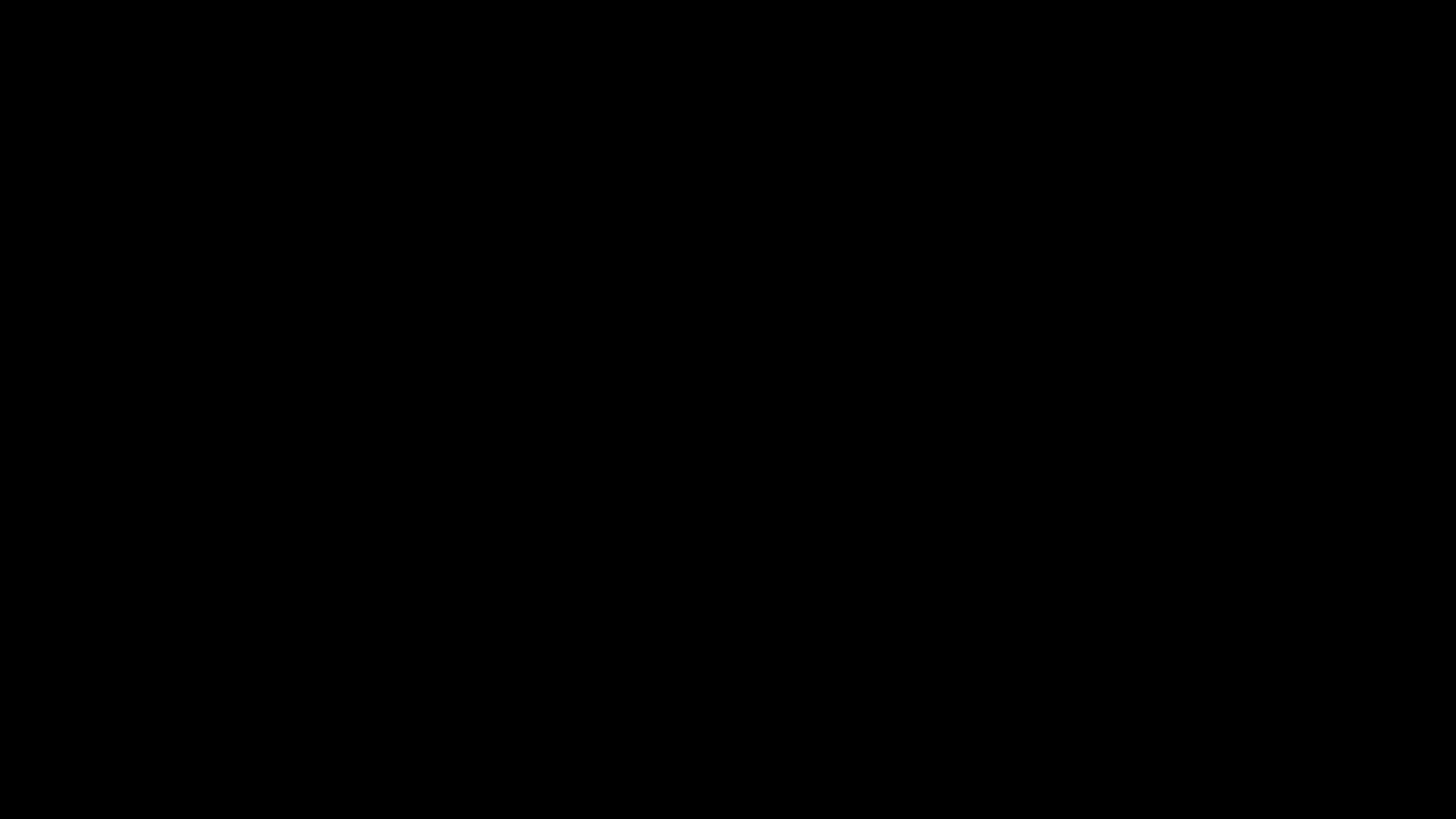


**Video S1.** Showing the behavior of a green sea turtle, *Chelonia mydas* during a ‘*G. divaricata* only’ (no-choice) feeding trial. The turtle was given a portion of the allelopathic, partially calcified red alga *Galaxaura divaricata*. After initial visual inspection and brief probing of the alga with its mouth the turtle refrains from feeding on *G. divaricata* for the rest of the trial.

**
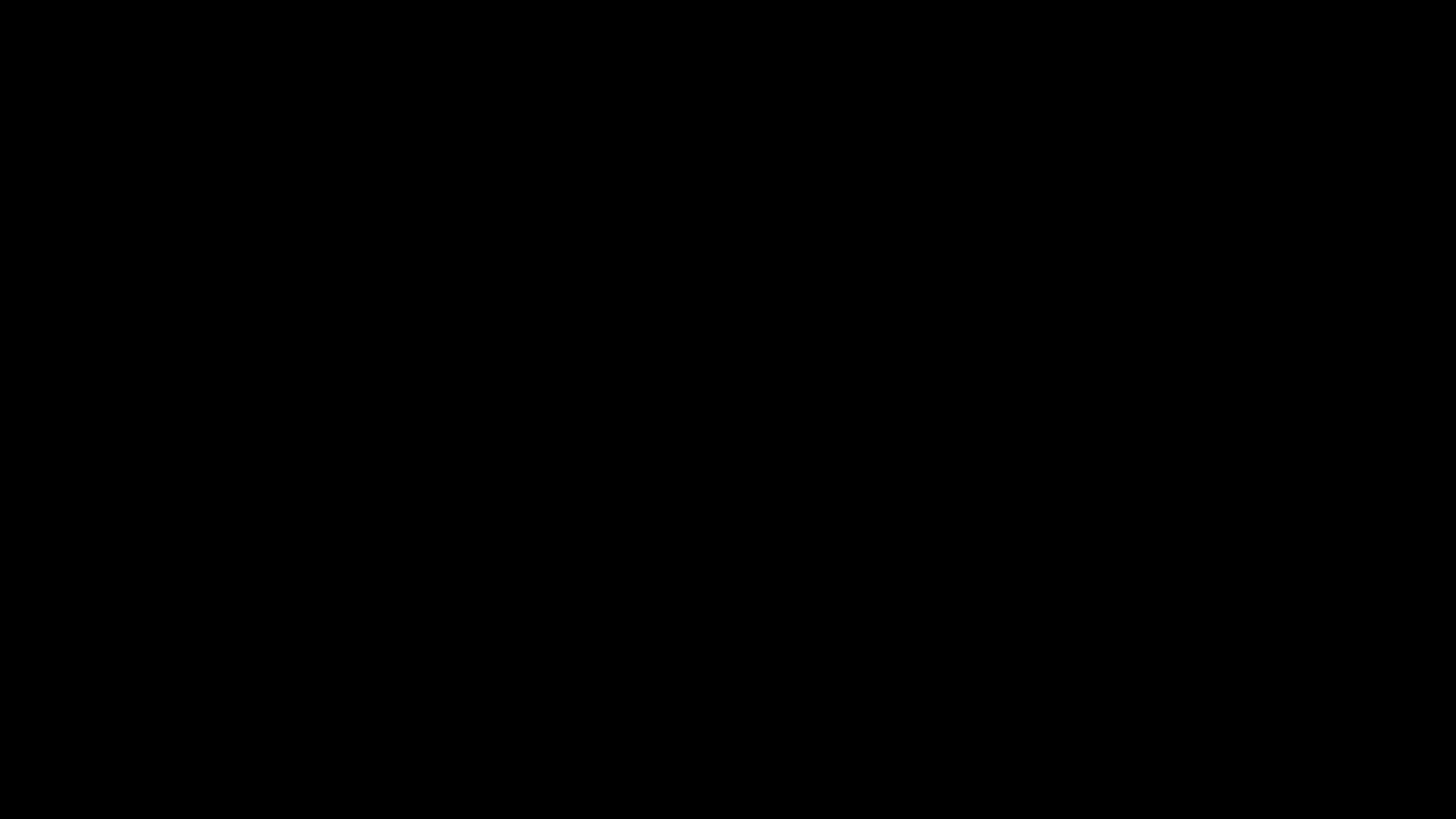
**

**Video S2.** Showing the feeding behavior of a green sea turtle, *Chelonia mydas* during a ‘mix’ algae treatment feeding trial. The turtle was given the partially calcified, allelopathic alga *Galaxaura divaricata* (dark red/brown) mixed with the fleshy, non-allelopathic alga *Gracilaria edulis* (light green/yellow). The turtle spends considerable time “cherry picking” pieces of *G. edulis* from the mix until there is only *G. divaricata* left in the tank. Sometimes the turtle picks the mixed algae portion up with its mouth and shakes it to separate the two algae species. It then continues to feed on *G. edulis* only*,* avoiding *G. divaricata.*
